# Supplementary material for: The POU-Domain Transcription Factor Oct-6/POU3F1 as a Regulator of Cellular Response to Genotoxic Stress
Source: Cancers (Basel). 2019 Jun 11;11(6):810. doi: 10.3390/cancers11060810 (PMC6627474; doi:10.3390/cancers11060810)
Supplement: Supplementary file 1 [file cancers-11-00810-s001.pdf]

Supplementary Materials

The POU-Domain Transcription Factor Oct-6/POU3F1 as a Regulator of Cellular Response to Genotoxic Stress

Cinzia Fionda, Danilo Di Bona, Andrea Kosta, Helena Stabile, Angela Santoni and Marco Cippitelli

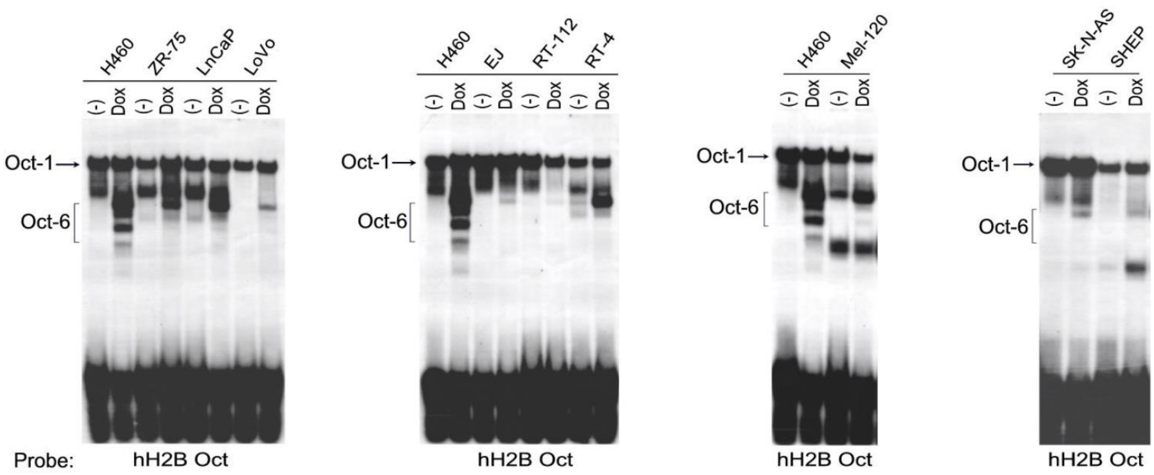

Figure S1. Doxorubicin induces Oct-6 DNA binding in different cancer cell lines. EMSA was performed using the hH2B-Oct probe in the presence of nuclear extracts (10 μg) from H460 cells (positive control for each panel) or from the indicated cell line, unstimulated (−) or treated with Dox (0.5 μM) for 24 h. DNA binding complexes with electrophoretic mobility corresponding to Oct-1 or Oct-6 are indicated in the figures.

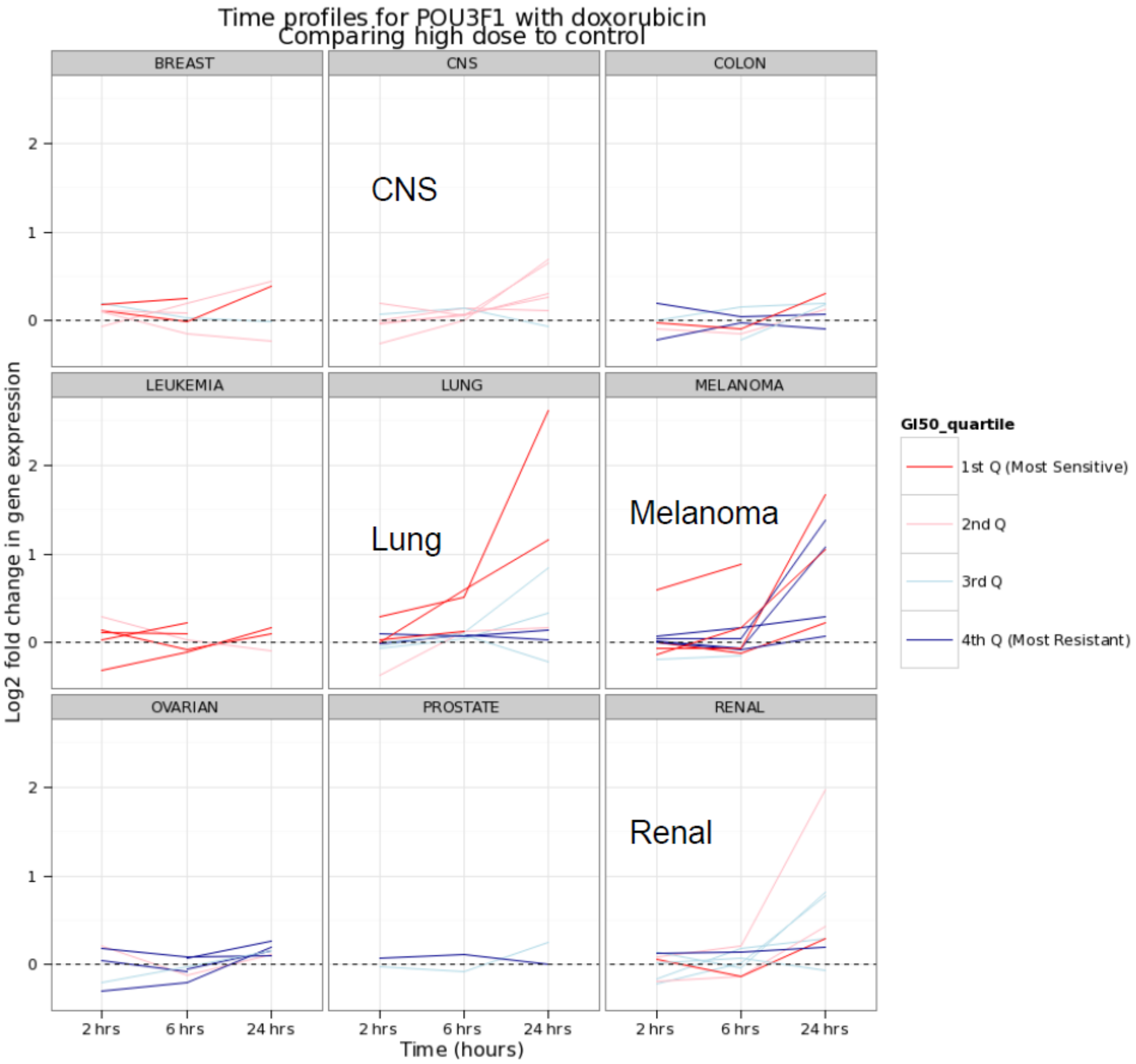

Figure S2. Time profiles for Oct-6 gene expression in different cell line lineages. Data shown in the picture were obtained using the interactive NCI Transcriptional Pharmacodynamics Workbench (NCI TPW) (https://tpwb.nci.nih.gov/GeneExpressionNCI60/index.html).

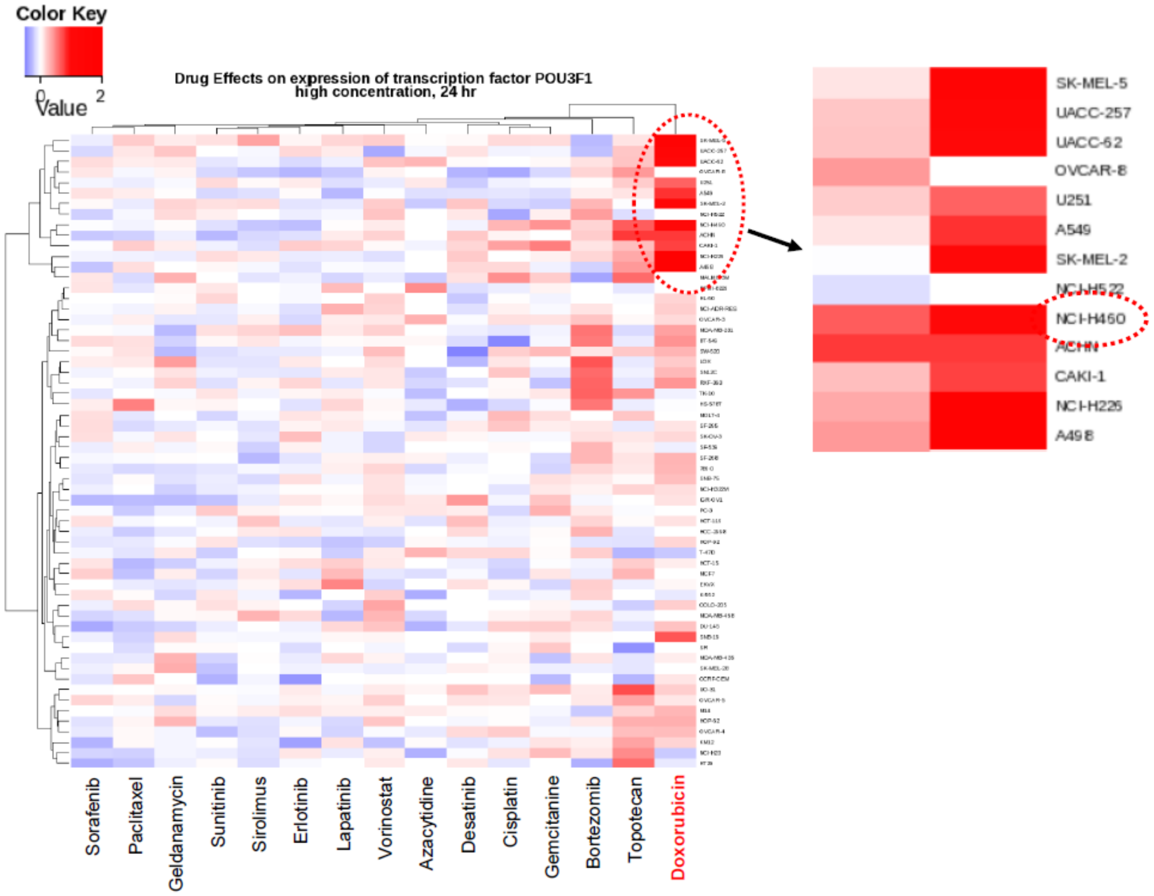

Figure S3. Heatmap of drug effects on expression of the transcription factor Oct-6: profiles in different cell line lineages treated with Dox. Expression of Oct-6 in the presence of 1 μM Dox/24 h was evaluated in selected human cancer cell lines as indicated in the red circles. NCI Transcriptional Pharmacodynamics Workbench. In the red circle, heatmap of drug effects on expression of a specific transcription factor (POU3F1/Oct-6) on selected human cancer cell lines available at (https://tpwb.nci.nih.gov/GeneExpressionNCI60/index.html).

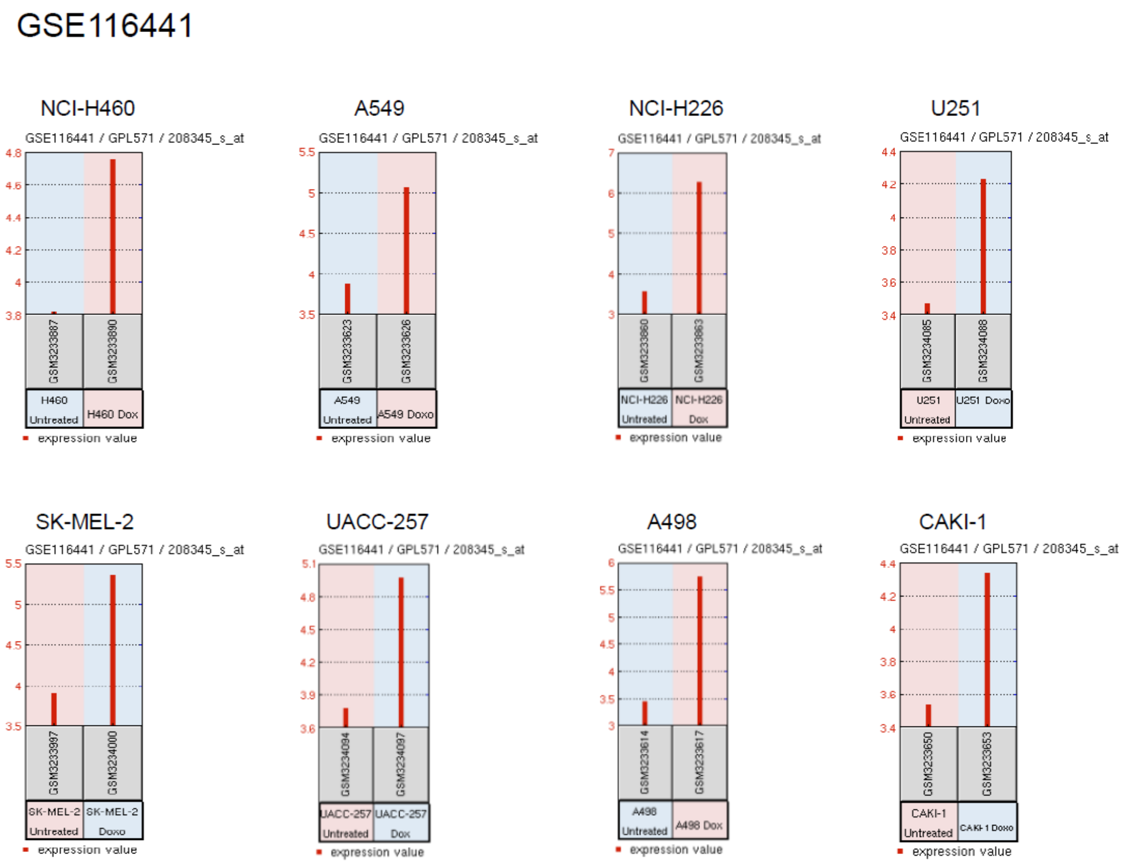

Figure S4. GEO2R analysis of the microarray public dataset GSE116441 available at (http://www.ncbi.nlm.nih.gov/geo/) for Oct-6 expression in selected cancer cell lines [(lung H460, A549, H226)–(melanoma SK-MEL-2, UACC-257)–(renal A498, CAKI-1)–(glioblastoma U251)] after treatment with Dox 1 µM for 24 h.

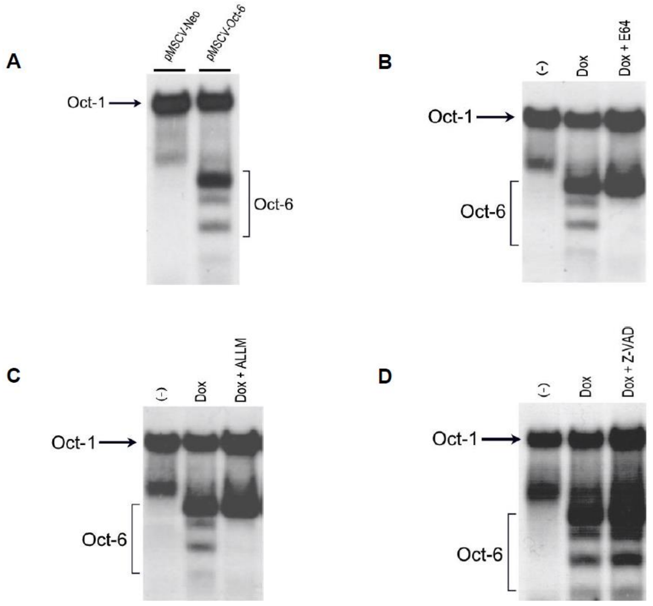

Figure S5. Characterization of Dox induced Oct-6 binding complexes. (**A**) EMSA showing the constitutive expression of Oct-6/Tst-1 in H460 cells stably transduced with the retroviral expression vector pMscv-Oct-6 or the control vector pMscv-Neo. (**B–D**) EMSA was performed using the ^{32}P-labeled hH2B-Oct probe in the presence of nuclear extracts (10 µg) from unstimulated (−) or Dox treated H460 cells (24 h) in the absence or in the presence of E64 (100 µM), ALLM (25 µM) or Z-VAD (10 µM). DNA binding complexes containing Oct-1 or Oct-6 are indicated in the figure. Data are representative of two independent experiments.

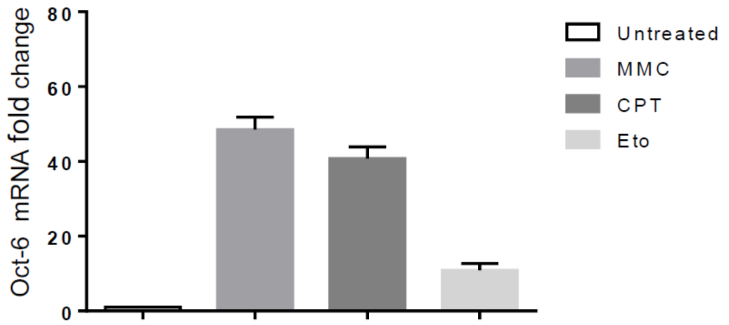

Figure S6. Different genotoxic drugs induce Oct-6 mRNA expression in H460 cells. Real Time PCR analysis of total mRNA obtained from H460 cells, untreated or treated with Mitomycin C 10 μM (MMC), Cisplatin 5 μg/ml (CPT) or Etoposide 10 μM (Eto) for 24 h. Data, expressed as fold change units, were normalized with GAPDH. A representative experiment of two is shown.

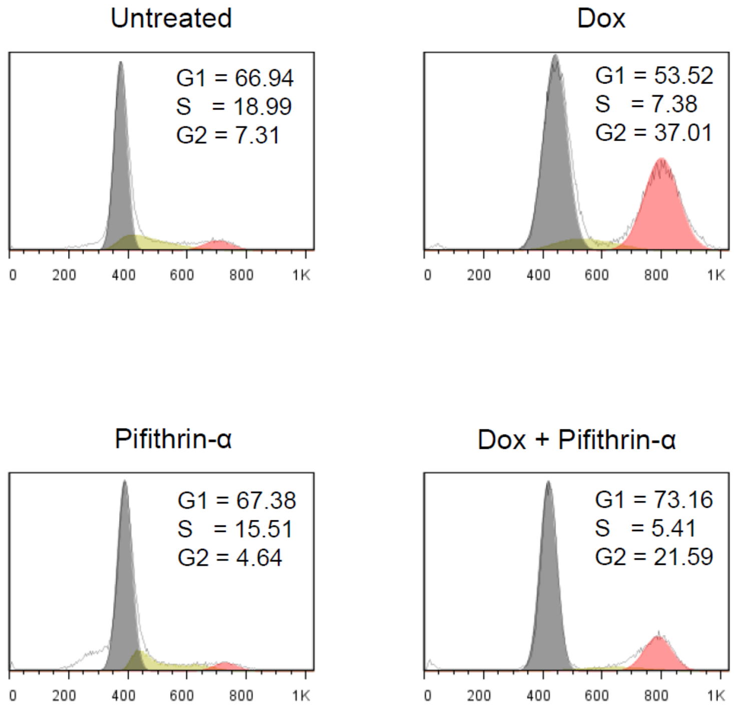

Figure S7. Cell-cycle analysis of H460 cells treated with Dox in the presence of Pifithrin-α. H460 cells, left untreated (−) or treated with Dox 0.5 μM in the presence or in the absence of Pifithrin-α (30 μM) for 24 h, were fixed and stained with PI to analyze cell distribution among the different cell cycle phases. A representative experiment is shown.

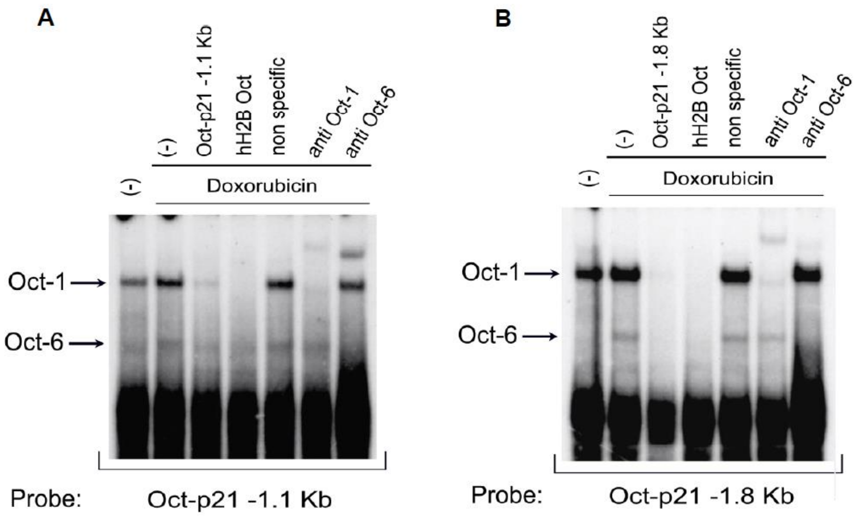

Figure S8. Oct-6 regulates p21 expression: direct binding to p21 promoter. EMSA was performed using two different consensus Octamer sequences from p21 promoter [1.1 Kb (left panel) (**A**) and -1.8 kb (right panel) (**B**)] as probe, in the presence of nuclear extracts (10 μg) from unstimulated (−) or Dox treated H460 cells for 24 h. Where indicated, 100 ng of specific consensus Oct or non-specific cold competitor was added to the reaction mixture to confirm specificity. Where indicated, anti-Oct-1 or anti-Oct-6 antibody was added to the reaction mixture. DNA binding complexes containing Oct-1 or Oct-6 are indicated in the figures. EMSA shown in the figure are representative of 3 independent experiments, all displaying similar results.

© 2019 by the authors. Licensee MDPI, Basel, Switzerland. This article is an open access article distributed under the terms and conditions of the Creative Commons Attribution (CC BY) license (http://creativecommons.org/licenses/by/4.0/).
